# Highlighting the Role of Mental Fatigue as a Health Risk Factor: A Narrative Review

**DOI:** 10.3390/sports14030106

**Published:** 2026-03-09

**Authors:** Jesús Díaz-García, Steven R. Bray, Tomás García-Calvo, Luca Bovolon, Marika Berchicci, Christopher Ring

**Affiliations:** 1 Faculty of Sport Sciences, University of Extremadura, 10003 Caceres, Spain; tgarciac@unex.es; 2 Department of Kinesiology, McMaster University, Hamilton, ON L8S 4L8, Canada; sbray@mcmaster.ca; 3 Department of Psychology, University G. d’Annunzio, 66013 Chieti-Pescara, Italy; luca.bovolon@phd.unich.it (L.B.); marika.berchicci@unich.it (M.B.); 4 Behavioral Imaging and Neural Dynamics (BIND) Center, University G. d’Annunzio, 66013 Chieti-Pescara, Italy; 5 School of Sport, Exercise and Rehabilitation Sciences, University of Birmingham, Birmingham B15 2TT, UK; c.m.ring@bham.ac.uk

**Keywords:** cognitive fatigue, fatigability, fall, sedentarism, Brain Endurance Training

## Abstract

Mental fatigue is a psychobiological state induced by sustained effortful cognitive efforts during daily life activities. Yet research efforts in exercise science have focused primarily on performance implications for athletes to the point of exclusion of vulnerable populations for which mental fatigue may be a health risk. This narrative review aims to clarify the role of mental fatigue on population health. Evidence suggest mental fatigue predisposes people to acute events related to temporary performance impairments (e.g., falls), and chronic diseases related to sedentarism (e.g., stroke, diabetes), as mental fatigue de-motivates people to engage in physical activity. Major risks are experienced by people with higher fatigability (i.e., people for whom mental fatigue is induced by less effortful tasks) and lower performance capacity. However, the few available information about moderators of fatigability and the lack of a normative protocol to assess mental fatigue are limiting the prevention of mental fatigue. Several strategies are used to counter mental fatigue acutely (e.g., caffeine ingestion); however, enduring countermeasures intended to alter psychobiological sequelae of mental fatigue, such as Brain Endurance and other trainings, are the only proved long-term countermeasures for mental fatigue. Yet the effectiveness of these interventions should be tested in populations with major risk for mental fatigue. We present a model identifying putative pathways through which mental fatigue may contribute to health risks to guide future investigations seeking to (a) evaluate the role of mental fatigue as a threat to health and well-being and (b) design interventions to mitigate the effects of mental fatigue in vulnerable populations.

## 1. Introduction

The COVID-19 pandemic highlighted the role of mental issues in health, including mental fatigue, defined as a psychobiological state induced by sustained cognitively demanding tasks [1]. Evidence shows that mental fatigue impairs physical [2] and cognitive performance capacity [3,4], and demotivates people from participating in physical activity or other leisure (daily life) activities that imply any physical effort [5,6], thus increasing their vulnerability to injuries and health risks (e.g., sedentarism or mental health issues). These effects are particularly concerning in vulnerable populations, such as older adults, individuals with chronic disease, or those with reduced cognitive or physical reserve, who may exhibit greater susceptibility to mental fatigue and adverse health outcomes. Despite its relevance, there has been an unfortunate paucity of research regarding mental fatigue in health research, particularly its role as a population-level health risk factor [7]. Most existing studies have focused on acute performance outcomes in young and healthy individuals, with limited integration of mental fatigue into broader public health and disease-prevention frameworks.

**Figure 1:**
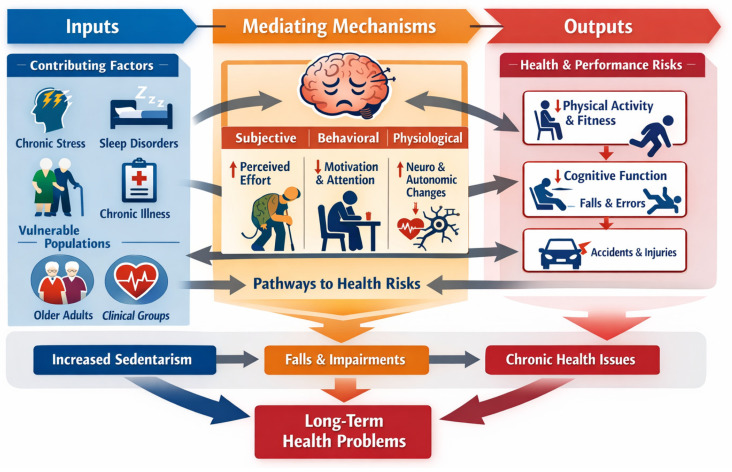
Conceptual model for mental fatigue as a population-specific health risk.

The objective of this narrative review is to critically synthesize existing evidence to conceptualize mental fatigue as a population-specific health risk factor, identify vulnerable groups, clarify underlying mechanisms, and discuss the implications for health, safety and disease prevention. To address this fundamental shortcoming and guide future research, we have drawn from the broader literature on mental fatigue to develop a conceptual mechanistic model: “The conceptual model for mental fatigue as a population-specific health risk” (see Figure 1). This figure is intended to provide a high-level visualization of the proposed pathways linking mental fatigue to health risks, facilitating understanding for a broad readership. This model has three interconnected levels: (1) inputs: factors contributing and moderating mental fatigue, (2) mediating mechanisms affected by mental fatigue, and (3) outputs: mental-fatigue-related impairments. For each level, explanation, literature gaps and future considerations are presented below.

## 2. Conceptual Model for Mental Fatigue as a Health Risk Factor

### 2.1. Inputs: Factors Contributing to Mental Fatigue and Fatigability

Common daily life experiences (e.g., driving in congested traffic, coping with personal problems, health issues, or work stress) have inherent cognitive demands that make people susceptible to mental fatigue [8]. In laboratory settings, mental fatigue is commonly induced using sustained cognitively demanding tasks (e.g., incongruent Stroop, AX-continuous performance task, or dual n-back). Once mental fatigue is induced, symptoms (e.g., increased feelings of tiredness, acute cognitive impairments, or demotivation for effort) and risks (e.g., falls, emotional dysregulation, road accident) have been reported to persist for at least 60 min [9] and up to several hours [10].

Mental Fatigability Theory [11] provides a useful framework to understand inter-individual susceptibility to mental fatigue. According to this theory, the level of mental fatigue induced by a given cognitive task varies across individuals due to moderators such as age, multimorbidity, lifestyle, sleep quantity and quality, and baseline cognitive performance. Fatigability has been shown to increase (i.e., same task = higher mental fatigue) with sedentarism [12], certain diseases such as cancer or CVD [11,13] and, also, ageing [14]. These populations represent vulnerable groups in whom identical cognitive demands may produce disproportionately greater mental fatigue and associated risks. In addition to clinical and ageing populations, athletes exposed to sustained cognitive and physical loads represent a specific population in which mental fatigability may be especially relevant. In high-performance sport contexts, repeated exposure to training-related cognitive demands (e.g., tactical decision-making, attentional control, emotional regulation) combined with high physical loads may exacerbate mental fatigue accumulation. Therefore, athletes—particularly those exposed to congested competition schedules or high cognitive demands—can also be considered a special population within the mental fatigue framework. However, there are empirical gaps that must be addressed to adequately assess these hypothesized effects:

Gap 1: The moderating role of cognitive performance and, especially, cognitive endurance in mental fatigue has not been studied, limiting the understanding of fatigue, and the design of appropriate targeted strategies for its prevention and mitigation.

Gap 2: The lack of a normative protocol for quantification of mental fatigue is limiting the development of regular large-scale studies for population’s mental fatigue and fatigability and, thus, the prevention of its associated risks. By identifying subjects with higher fatigability or detecting particular events that induce exacerbated levels of mental fatigue, experts and stakeholders would be able to design targeted strategies to better prevent mental-fatigue-related acute and chronic health risks.

### 2.2. Mediating Mechanisms

Mental fatigue impairs performance and health through subjective, behavioral, and physiological mechanisms [2].

At the subjective level, mental fatigue consistently increases the Rate of Perceived Effort (RPE) for a given task, compared with non-fatigued conditions [1,2]. This increase in perceived effort occurs despite unchanged cardiovascular and metabolic markers, representing one of the most robustly demonstrated mechanisms of mental fatigue [2]. This dissociation between subjective effort and physiological strain is critical, as it explains why mentally fatigued individuals voluntarily reduce physical activity, thereby increasing sedentary behavior and health risks.

At the behavioral level, mental fatigue induces demotivation and reduced willingness to engage in effortful activities [5]. This may be related to impaired attention control and greater difficulty selecting relevant stimuli [15]. In athletes, mental fatigue has been shown to impair decision-making processes, while in non-athlete populations it may contribute to reduced participation in social and leisure activities, increased accident risk, and poorer adherence to health-promoting behaviors.

At the physiological level, evidence remains limited. Mental fatigue has been associated with alterations in neural network efficiency, autonomic regulation, and parasympathetic activity [3,4], but findings remain inconsistent. Methodological constraints, including high costs and technical complexity, have hindered progress in identifying robust physiological markers of mental fatigue and fatigability.

Gap 3: Knowledge about mechanisms underlying mental fatigue and fatigability, especially physiological ones, remains limited in this research topic. Clarifying the mechanisms underlying mental fatigue is essential for understanding its phenomenology and the development of adequate—targeted—strategies for its prevention and mitigation (e.g., improving fatigability).

### 2.3. Outputs: Health and Performance Consequences

Mental fatigue worsens endurance performance and engagement in physical activity [2]. Increased perception effort leads to reductions in exercise duration [1], intensity and training volume [16], which may attenuate health benefits of physical activity and promote sedentary behavior. In athletes, these mental-fatigue-related performance decrements may have additional implications, as chronically elevated perceived effort and reduced training tolerance can contribute to maladaptive training responses. Specifically, sustained mental fatigue may increase the risk of non-functional overreaching or overtraining by impairing the regulation of training load, recovery, and decision-making related to effort allocation. Over time, this may predispose athletes to injury, burnout, and prolonged performance decline, reinforcing the relevance of mental fatigue not only as a performance-limiting factor but also as a health-related risk within high-performance sport environments. Importantly, these fatigue-induced changes in behavior are not mediated by changes in cardiovascular and metabolic systems [17], which means those systems are not challenged to the point where adaptations would occur when people are mentally fatigued. Mental fatigue, therefore, reduces the potential health benefits of exercise training and predisposes the population to not achieving the minimum physical activity recommendations, which in turn increases the health risks associated with a sedentary lifestyle, such as cardiovascular problems, different types of cancer, diabetes, and depression, among others [18]. The lack of attendance to social (e.g., visit to friends or parents), leisure activities, or other events that imply some levels of physical activity (e.g., homework, walking) due to a mental fatigue-related increase in RPE may also contribute to the development of episodes of anxiety and depression, with mental health issues being recognized as a pandemic in the XXI century.

Mental fatigue is also shown to worsen cognitive performance [4]. While there has been much interest of researchers on the effects of mental fatigue on physical performance, less studies have investigated the effects of mental fatigue on cognitive performance. However, latest evidence shows that mental fatigue can cause transient impairments on cognitive performance, which increases the risk of falls [19,20] and impairs decision-making [21], predisposing people to accidents [22]. Falls represent a major public health concern as a leading cause for functional disability and loss of independence, particularly among older and disease populations, while accidents are among the leading causes of mortality among youth and non-disease populations. Future programs aiming at their prevention should originally take mental fatigue into account, among the causes contributing to both phenomena. Together, these findings heighten concerns that people who may already experience impairments in physical and cognitive performance, such as older adults, are at higher risk of developing diseases, having accidents, and other health problems associated with mental fatigue [19,23]. Besides this information:

Gap 4: The effects of mental fatigue on physical performance and cognitive functions have been mainly tested only with athletes and healthy young adults, with less available information on the effects of mental fatigue among older adults and other vulnerable populations such as mild cognitive impairment, dementia, or cancer patients.

Importantly, the recognition of adverse outputs related to mental fatigue highlights the necessity of adopting approaches aimed at mitigating mental fatigue across different contexts, including occupational, clinical, and everyday settings. Addressing mental fatigue should be considered a priority not only to prevent acute incidents, such as accidents and falls, but also to reduce long-term health risks associated with physical inactivity, lack of participation in leisure activities and functional decline. Moreover, the increasing cognitive demands of modern lifestyles and work environments suggest that the prevalence and impact of mental fatigue are likely to continue rising, further amplifying their health and economic burden. Therefore, integrating mental fatigue as a modifiable factor within health promotion, injury prevention, and safety frameworks is essential, especially in vulnerable populations. Emphasizing the importance of mitigating mental fatigue may facilitate the development of more comprehensive health strategies, support safer behaviors, and ultimately contribute to improved health outcomes and reduced strain on healthcare systems. Below, a comprehensive overview of the most effective tested practical strategies for mental fatigue is detailed.

## 3. Strategies for Mental Fatigue Mitigation

Short-term strategies such as listening to motivating music, ingesting caffeine, and provision of extrinsic motivation or rewards have been shown to be effective in mitigating the acute impact of mental fatigue on performance, while, in the long term, cognitive and physical training interventions show effectiveness in improving fatigability, reducing mental fatigue and mitigating the detrimental effects of mental fatigue on performance [24].

Regarding acute strategies for mitigating the effects of mental fatigue, several interventions have demonstrated measurable improvements in performance and subjective indicators of mental fatigue. Caffeine ingestion has been shown to enhance endurance performance in mentally fatigued individuals; for example, in a cycling protocol following a mental fatigue induction, caffeine increased time to exhaustion by approximately 14% compared with placebo, indicating a significant mitigation of the deleterious effects of mental fatigue on physical performance and perceived effort [25]. Caffeine, for example, may act through antagonism of adenosine receptors, enhancing dopaminergic signaling and reducing perceived effort, thereby partially restoring performance under mental fatigue conditions. Likewise, broader meta-analytic evidence suggests overall endurance and time-to-exhaustion improvements of roughly 15–17% with moderate caffeine doses in endurance contexts, underscoring its acute ergogenic and anti-fatigue potential [26]. Daytime napping also produces quantifiable benefits, while the exact percentage reductions in fatigue vary by task and nap duration [27]. Finally, although the percentage effects of exposure to natural environments are less uniformly reported, experimental work has found that cognitive task performance (e.g., memory and attention) can improve by approximately 20% after walks in natural settings compared with more urban or cognitively demanding environments, signifying restorative effects on attentional resources depleted by mental fatigue [28].

Long-term strategies are especially promising because they have the potential to improve fatigability, enhance mental fatigue resilience and reduce susceptibility to physical and cognitive decline under sustained cognitive demands. While physical and cognitive training have independently been shown to produce improvements in people’s cognitive performance, Brain Endurance Training (BET), a novel training system that combines physical and cognitive tasks, appears to be another promising intervention for preventing mental fatigue and its risks [29]. BET produces greater benefits in physical and cognitive performance than either physical or cognitive training alone [30,31] by combining the benefits of both types of training. In trained road cyclists, Staiano et al. [29] demonstrated that six weeks of BET resulted in substantial improvements in endurance performance when mentally fatigued. Specifically, time-to-exhaustion during constant-load cycling tests increased by approximately 11–17% in the BET group, compared with marginal improvements of around 2–3% in control groups performing physical training alone. Recent randomized clinical trials in older adults with mild cognitive impairment have also shown that exercise combined with cognitive training leads to larger improvements in cognitive outcomes than exercise alone, with medium effect sizes (Cohen’s d ≈ 0.58–0.65) for global cognition scores. Importantly, BET shares conceptual similarities with Dual-Task Training, which has been widely applied in older adults and clinical populations to address cognitive–motor interference. The key distinction lies in BET’s systematic and progressive induction of mental fatigue, explicitly targeting effort perception and fatigue tolerance, whereas traditional Dual-Task Training often focuses on task coordination rather than fatigue resilience. Integrating insights from both approaches may enhance intervention design for vulnerable populations.

The hypothesis that cognitive performance moderates or influences mental fatigability supports the affirmation that BET generates resilience against mental fatigue. That is, it creates adaptations within individuals to maintain performance and reduce the risk of injury in situations where they experience mental fatigue [32,33]. BET training is designed to systematically induce mental fatigue using cognitive stimuli, and increases the perception of effort, so repeated exposure to BET improves the regulation of effort perception in the presence of mental fatigue, helping to maintain performance. In the previously mentioned study of Staiano et al. [29], BET has been shown to improve cognitive outcomes under fatigue. Participants undergoing BET demonstrated faster reaction times and improved performance in executive function tasks, suggesting enhanced cognitive control when mentally fatigued. These findings support the notion that BET improves the ability to sustain cognitive performance under prolonged demands. In line, Díaz-García et al. [32] showed that BET improved and preserved neuromuscular performance during mentally fatigued conditions, with participants exhibiting significantly smaller decrements in chest press strength and squat jump height compared with control training. Across tasks, performance maintenance ranged from approximately 8–12% higher in the BET condition when mental fatigue was present. Importantly, these performance gains occurred without significant changes in traditional physiological markers, indicating that BET primarily influenced central factors related to effort perception and fatigue tolerance. However:

Gap 5: The benefits of short- and long-term strategies to reduce mental fatigue, its effects and risks, have been mainly tested in young adults and athletes, with less information for its effectiveness to mitigate mental fatigue in populations with higher fatigability and higher mental-fatigue-associated risks such as older and disease populations.

## 4. Future Research

Despite the growing body of evidence identifying mental fatigue as a relevant health risk, several important knowledge gaps remain and should guide future research in this field. First, the moderating role of cognitive performance—and particularly cognitive endurance—on mental fatigue and fatigability has received little attention. Most studies have focused on the acute effects of mentally fatiguing tasks on performance, without considering interindividual differences in capacity to sustain cognitive effort over time [34]. Understanding how baseline cognitive abilities and cognitive endurance influence development, magnitude, and consequences of mental fatigue is essential to better characterize vulnerable profiles and to design targeted prevention and mitigation strategies tailored to individual capacities.

Second, progress in this research area is limited by the lack of a standardized, normative protocol for the quantification of mental fatigue [35]. Current approaches rely on heterogeneous cognitive tasks, subjective scales, or indirect performance outcomes, which hampers comparability across studies and limits feasibility of large-scale epidemiological investigations. The development and validation of a robust, standardized assessment framework would enable population-level monitoring of mental fatigue and fatigability. Such tools could help identify individuals with heightened susceptibility or detect contexts and events that induce exacerbated mental fatigue, thereby supporting stakeholders and health professionals in implementing early, targeted interventions to reduce acute and chronic health risks associated with mental fatigue.

Third, knowledge of the mechanisms underlying mental fatigue and fatigability—particularly physiological mechanisms—remains incomplete [36,37]. While psychological and behavioral models have provided valuable insights, there is still limited understanding of how central and peripheral physiological processes (e.g., neural activation patterns, neurotransmitter dynamics, autonomic regulation, inflammatory responses, or cerebral oxygenation) contribute to the onset and progression of mental fatigue. Clarifying these mechanisms is crucial for advancing phenomenological understanding of mental fatigue and for informing the development of mechanism-based, targeted strategies aimed at improving fatigability and resilience.

Fourth, existing evidence on the effects of mental fatigue on physical performance and cognitive function has been largely derived from studies on athletes and healthy young adults. Consequently, generalizability of current findings to older adults and other vulnerable populations—such as individuals with mild cognitive impairment, dementia, cancer, or other chronic conditions—remains limited [38,39]. Given that these populations may exhibit higher fatigability and greater susceptibility to adverse consequences of mental fatigue, future studies should prioritize inclusive designs that explicitly examine age- and disease-related differences in mental fatigue responses and outcomes. While athletes have traditionally been the primary population of interest in mental fatigue research, future investigations should explicitly distinguish between performance-oriented outcomes and health-related consequences. In particular, studies should examine how chronic exposure to mental fatigue interacts with training load, recovery, and cognitive demands to influence maladaptive outcomes such as overtraining, injury risk, and long-term well-being in athletic populations.

Finally, although several short- and long-term mitigation strategies (e.g., cognitive training, physical exercise, combined interventions) show promise, their effectiveness has been tested predominantly with young, healthy, and athletic populations. There is a critical need to evaluate whether these strategies are equally effective, feasible, and safe in populations with higher baseline fatigability and elevated mental-fatigue-related health risks, such as older adults and clinical populations [40]. Addressing these gaps will be essential for translating current knowledge into effective, equitable, and population-specific approaches for preventing and mitigating mental fatigue and its associated health consequences.

## 5. Conclusions

Mental fatigue represents a relevant and underrecognized contributor to adverse health outcomes, increasing both acute risks (e.g., accidents, falls) and chronic risks through reduced physical activity and social participation. The lack of standardized assessment tools and limited understanding of fatigability constrain prevention efforts. Cognitive, physical, and combined interventions such as BET and dual-task approaches show promise, but further research is required in vulnerable populations.

## Data Availability

No new data were created or analyzed in this study.
